# Occurrence and Antibiotic Resistance Risk Burden of *Vibrio mimicus* Isolates from Seafood and Aquatic Environments

**DOI:** 10.3390/antibiotics14111075

**Published:** 2025-10-26

**Authors:** Temitope C. Ekundayo, Frederick T. Tabit

**Affiliations:** Department of Life and Consumer Sciences, University of South Africa, Private Bag X6, Florida, Roodepoort 1710, South Africa

**Keywords:** *Vibrio mimicus*, antibiotic resistance, one-health, seafoods, wastewater, public health, shrimps, rivers

## Abstract

Background: Emerging antimicrobial resistance in *Vibrio mimicus* (Vm) associated with seafood may exacerbate infections in patients. Method: This study investigated the prevalence of antibiotic resistance and its cross-sample/territory risk burden in Vm from seafood and aquatic environment using hierarchical mixed-effects and antimicrobial resistance risk index (ARRI) modelling. Results: Among the Vm isolates, resistance was highest to amoxicillin (83.7%, 5.3–99.8) and streptomycin (54.6%, 95% CIs: 15.8–88.5), with generally high resistance to penicillins (58.0–98.0%), macrolides (17.2–65.8%), and colistin sulphate (80.2%). Resistance to aminoglycosides, cephalosporins, tetracyclines, and fluoroquinolones varied widely, with seafood and environmental water sources showing similar trends. Notably, resistance to nalidixic acid (47.2%, 17.3–79.4) and doxycycline (59.4%, 3.6–98.3) was prominent. Carbapenem resistance remained low, especially in seafood. Chloramphenicol resistance (32.3%, 2.7–89.0) was higher in environmental water. Trimethoprim–sulfamethoxazole resistance was relatively low (5.8%, 0.7–36.1). Ampicillin–sulbactam resistance (43.3%, 5.1–91.5) exceeded that of amoxicillin–clavulanic acid (31.8%, 0.8–96.3). The current data reveal antibiotic resistance burdens (ARBs) of Vm in seafood (ARRI ≈ 50) and waters (ARRI ≈ 46) exceeded that of human isolates (ARRI ≈ 0.01) greatly. Also, it identified Nigeria (ARRI = 7.78)/India (ARRI = 7.35) and Asia (ARRI = 56.91)/Africa (ARRI = 40.12) as hotspots of Vm ARBs. Conclusions: Overall, Vm exhibited diverse antimicrobial resistance patterns across sources with high resistance concerns and high rates against penicillins, cephalosporins, macrolides, and sometimes polymyxins. Thus, it is recommended that stricter regulations on antibiotic use in aquaculture are enforced; wastewater treatment is improved, one-health surveillance is implemented; and education of stakeholders about resistance risks, use of alternatives, and proper cooking of seafood to mitigate Vm-resistant impact is promoted.

## 1. Introduction

Antimicrobial resistance (AMR) is a critical global health threat, with recent estimates attributing 4.71 million deaths in 2021 to drug-resistant infections [1] Aquatic ecosystems are increasingly recognised as reservoirs and pathways for resistance genes [2,3]. *Vibrio* are marine and estuarine bacteria that often harbour diverse antimicrobial resistance (AMR) determinants for a broad spectrum of antimicrobials [4]. AMR against clinically important drugs is increasingly being documented and detected in *Vibrio* species from seafood and human infections, with resistance genes frequently located on mobile genetic elements [2]. Thus, aquatic environments serve as significant reservoirs for AMR genes, which can be transmitted to human pathogens in the food and water supply chain [2,3].

Vm, a close relative of *V. cholerae* that has been isolated globally from coastal and freshwater habitats, as well as seafood, can cause cholera-like gastroenteritis [5]. Unlike *V. cholerae*, Vm generally lacks cholera toxin; however, it can carry other virulence factors that have the potential to cause severe illness [5]. A recent outbreak in Florida (USA) linked raw seafood to Vm infection, where six persons fell ill with severe diarrhoea and four were hospitalised (one in intensive care) despite the strains being cholera-toxin-negative, confirming Vm as a foodborne disease threat [6]. This confirms that Vm can be a foodborne threat. In aquaculture, Vm also contributes to losses in shrimp, catfish, and other farmed species by death and high control cost, potentially acquiring AMR under antibiotic selection [3,5].

Seafood, which is widely farmed and consumed globally, constitutes a key vector for Vm exposure [7]. Antibiotics are commonly used in intensive shrimp aquaculture to control diseases, but this practice exerts selective pressure that promotes the emergence and proliferation of antibiotic-resistant bacteria [8]. In a study of retail shrimp in California, *Vibrio* spp. was detected in 60.25% of samples, with approximately 47.25% of these isolates exhibiting ampicillin resistance; whole-genome sequencing further revealed dozens of unique antimicrobial resistance (AMR) genes distributed across multiple *Vibrio* species [7]. Whole-genome sequencing of shrimp-derived *Vibrio* revealed dozens of unique AMR genes spread across multiple species [7]. These findings illustrate how commonly consumed seafoods such as shrimps, oysters, and other bivalves can harbour *Vibrio* pathogens and AMR genes [9]. Pacific oysters, for instance, have been found to contain antibiotic-resistant bacteria carrying various AMR genes including beta-lactamases [10]. Findings from laboratory studies have confirmed that oysters can accumulate both *Vibrio* pathogens and AMR genes, underscoring their potential as vectors of AMR genes [10]. In general, consumption of raw or minimally cooked shellfish, such as oysters, clams, mussels, amongst others, has long been associated with *Vibrio* infections in which diseases can be exacerbated by the presence of antibiotic-resistant genes [2,10]. Other seafoods such as fish, crab, and shrimp, amongst others, are common sources of *Vibrio* species, with surveys in Europe and North America reporting prevalence rates exceeding 50–60% [7,11,12].

The combination of Vm pathogenicity and AMR presents significant food safety and public health concerns, with infections resembling cholera-like disease, characterised by profuse diarrhoea [2,6]. AMR could compound Vm infection especially in cases where ampicillin, which is often used empirically to treat vibriosis, may become ineffective due to ampicillin resistance [2,7]. Environmental contamination poses a significant threat to human health, as *Vibrio* species persist in coastal and freshwater ecosystems where human activities—such as sewage discharge and agricultural runoff—introduce both pathogens and antibiotics into water systems. Furthermore, conventional surface and drinking water treatments do not consistently remove antibiotic residues or antibiotic-resistant bacteria, allowing these hazards to persist and potentially spread [3].

The horizontal transfer of AMR genes plays a critical role in the spread of antimicrobial resistance among *Vibrio* species, considering that plasmids, integrons and transposons, which carry AMR genes, can easily be exchanged [13,14]. Despite these concerns, significant gaps remain in the understanding of antimicrobial resistance (AMR) in Vm, as reports are sparse and few studies have systematically catalogued the AMR profiles of these species. Most research studies have focused on AMR in *V. cholerae*, *V. parahaemolyticus,* and *V. vulnificus* [2]. The rising incidence of antibiotic-resistant Vm in seafood and aquatic environments has necessitated a study on the global distribution of AMR in one-health concern. Therefore, this aims to investigate the prevalence of AMR in Vm isolated from seafood and the aquatic environment, to identify key trends that necessitate enhanced surveillance and further research.

## 2. Results

### 2.1. General Data Overview

A total of 408 data sources were identified, of which 19 eligible sources were included in the final database for analysis (Figure S1). The summary details of the nineteen eligible sources are presented in Table S1a. They detailed antimicrobial resistance data of Vm isolates from seafoods including oyster, fish, crab, prawn, clam, mussel, lobster, shrimps, and cuttle fish [15,16,17,18,19,20,21,22,23,24,25,26,27]; environmental water [28,29,30,31,32,33]; and clinical samples [19]. Fifty-one different antibiotics including amikacin, amoxicillin–clavulanic acid, amoxicillin, ampicillin, ampicillin–sulbactam, azithromycin, cefotaxime, cefoxitin, ceftazidime, ceftriaxone, ceftriazone, cefuroxime, cefixime, cephalothin, carbenicillin, chloramphenicol, ciprofloxacin, colistin sulphate, polymyxin B, doxycycline, erythromycin, rifampicin, gatifloxacin, gentamicin, imipenem, kanamycin, levofloxacin, meropenem, netilmicin, nitrofurantoin, norfloxacin, nalixidic acid, ofloxacin, pefloxacin, penicillin, piperacillin, streptomycin, tetracycline, oxytetracycline, trimethoprim–sulfamethoxazole, trimethoprim, tobramycin, neomycin, sulfadiazine, sulfamethoxazole, sulfamethoxypyrimidine, imipenem, florfenicol, nitrofurantoin, oxolinic acid, and compound sulphonamides were tested against Vm isolates (Table S1).

#### Pooled Sample and Sample-Specific Prevalence of Different Antibiotic Resistance in *V. mimicus*

Table 1 presents pooled sample and sample type-specific prevalence of various antibiotic resistance in Vm isolates from seafoods, environmental, and clinical samples.

### 2.2. Aminoglycosides

The pooled resistance to aminoglycosides among the 423 isolates across pooled samples showed notable variation coupled with variable heterogeneity with the highest against streptomycin (54.6%, 95% CIs, 15.8–88.5), followed by kanamycin (28.8%, 3.5–81.9), amikacin (22.6%, 4.6–64.1), and least against gentamicin (16.5%, 2.8–57.4) (Table 1). Similar results were also obtained in seafood- and environmental water-specific Vm resistance prevalence against aminoglycosides. The test for subgroup differences in aminoglycoside antibiotic resistances between seafoods and environmental water isolates were significant except for amikacin (Figure S2). Streptomycin-resistant Vm in seafood and environmental water had 51.8% (9.5–91.7) and 79.3% (0.1–100.0) pooled prevalence, respectively. The pooled prevalence of kanamycin-resistant, gentamicin-resistant, and amikacin-resistant Vm isolates in seafoods was 50.3% (5.8–94.4), 26.7% (3.7–77.6), and 19.2% (2.0–73.7). Also, the pooled Vm streptomycin, amikacin kanamycin, and gentamicin resistance rates in environmental water were 79.3% (0.1–100.0), 30.8% (0.0–100.0), 25.1% (0.2–98.6), and 5.2% (0.1–80.4), respectively (Table 1 and Figure S2).

### 2.3. Carbapenems

The pooled meropenem resistance (12.3%, 1.6–54.6) was higher than the imipenem-resistance (10.7%, 2.5–35.9) among the 423 Vm isolates (Table 1 and Figure S2). While imipenem-resistant (2.1%, 0.0–99.9) and meropenem-resistant (6.6%, 0.0–100.0) Vm have relatively low pooled rates in seafood, imipenem-resistant (16.3%, 1.9–65.6) and meropenem-resistant (16.9%, 1.7–70.6) Vm were similar in environmental water (Table 1 and Figure S2). The test for carbapenem-resistant subgroup differences for seafood and environmental water Vm isolates were significant for meropenem and imipenem (Figure S3).

### 2.4. Cephalosporins

Among the 423 Vm isolates, cefuroxime-resistant Vm (60.1%, 2.3–99.0) had the highest pooled rate among the cephalosporins, followed by cephalothin (28.7%, 13.3–51.4) and cefotaxime (8.9%, 2.3–29.3) (Table 1 and Figure S4). However, cephalothin-resistant pooled rate in seafoods was 27.6% (9.3–58.5) while cefotaxime-resistant Vm pooled rate was 7.3% (2.3–21.13). The test for subgroup differences in cefuroxime-, cephalothin-, and cefotaxime-resistant rates in Vm isolates from seafood and environmental water were significant (Figure S4).

### 2.5. Chloramphenicol

The pooled chloramphenicol resistance among 423 Vm isolates was 32.3 (2.7–89.0). However, its pooled rate was higher in environmental water (51.9%, 0.0–100.0) compared with seafoods (20.8%, 1.1–85.9) (Table 1 and Figure S4). The test for chloramphenicol-resistance differences among Vm isolates from seafood and environmental water were significant (Figure S4).

### 2.6. Fluoroquinolones and Quinolones

Vm had variable pooled resistance rates among all-sample (423) and sample-specific isolates. Vm had the highest pooled resistance prevalence rate against nalixidic acid (47.2%, 17.3–79.4), then ofloxacin (34.0%, 1.7–94.0), ciprofloxacin (9.9%, 1.7–41.6), and the least was norfloxacin (8.9%, 0.4–70.9) among the 423 all-sample isolates (Table 1). In seafoods, nalixidic acid resistance was 39.7% (5.2–88.7), norfloxacin resistance was 18.0% (1.1–81.5), and ciprofloxacin resistance was 8.6% (0.7–56.4) among Vm isolates. For environmental water, the ciprofloxacin-resistant Vm pooled rate was 12.9% (0.1–97.3) while norfloxacin-resistant Vm pooled rate was 1.4% (0.0–100.0). Vm resistance rates to fluoro/quinolones were significantly different among seafood and environmental water isolates except against nalixidic acid (Figure S5).

### 2.7. Macrolides and Azalides

Azithromycin-resistant Vm had 65.8% (0.9–99.5) while erythromycin-resistant Vm has 21.5% (3.0–70.8) pooled rates among the 423 Vm isolates (Table 1). The pooled erythromycin Vm was 17.2% (4.3–49.2) in seafoods. The level of heterogeneity (51.7–72.2%) was generally low among the macrolides and azalides (Figure S3). Vm resistance rates to fluoro/quinolones were significantly different among seafood and environmental water isolates except against nalixidic acid (Figure S5).

### 2.8. Penicillins

Vm resistance against penicillins was generally high with amoxicillin resistance leading with a pooled rate of 83.7% (5.3–99.8), followed by penicillin (72.7%, 43.5–90.3), and ampicillin (61.11%, 24.5–88.4), among the all-sample Vm isolates (Table 1). Amoxicillin-resistant Vm (98.0%, 0.1–100.0) pooled rate was higher than ampicillin-resistant (76.1%, 17.2–98.0), and penicillin-resistant (72.7, 43.5–90.3) Vm isolates from seafoods. The pooled rate of environmental water-specific ampicillin-resistant Vm isolates was 58.0% (19.4–88.8). Figure S6 shows that Vm resistance rate was only significantly different among seafood and environmental water isolates for amoxicillin among penicillins.

### 2.9. Polymyxins and Sulfonamides

The pooled colistin sulphate resistance and sulfamethoxazole resistance rates among the isolates were 80.2% (0.0–100.0) and 38.3% (3.6–91.2), respectively, while seafood-specific sulfamethoxazole pooled prevalence was 25.8% (1.8–86.9) (Table 1). Sulfamethoxazole resistance rate was significantly different in seafood Vm isolates (Figure S7).

### 2.10. Tetracyclines

The pooled Vm resistance rate was variable among the doxycycline-resistant all-sample isolates leading with 59.4% (3.6–98.3), followed by oxytetracycline-resistant (47.9%, 26.1–70.5), and tetracycline-resistant (13.5%, 2.9–45.2) (Table 1) isolates. Vm isolates from seafoods had resistance to the tetracyclines such as oxytetracycline (52.9%, 20.3–83.3), doxycycline (36.5%, 0.0–100.0), and tetracycline (12.8%, 2.6–45.0) compared with isolates from environmental water (tetracycline, 35.1%, 1.2–96.0). Vm resistance rates were generally not significantly different among seafood and environmental water isolates among the tetracyclines (Figure S8).

### 2.11. Trimethoprim–Sulfamethoxazole

Trimethoprim–sulfamethoxazole-resistant Vm pooled rate was 5.8% (0.7–36.1), 19.0% (0.4–92.6), and 4.9% (0.4–41.0) in all-sample, environmental water, and seafoods, respectively (Table 1). However, the level of heterogeneity was higher in environmental water isolates (84.4%) compared with seafoods (0%) and all-sample (73.3%) isolates. The trimethoprim–sulfamethoxazole resistance rate was not significantly different among seafood and environmental water Vm isolates (Figure S8).

### 2.12. β-Lactam/β-Lactamase Inhibitor

The ampicillin–sulbactam-resistant Vm pooled rate (43.3%, 5.1–91.5) was higher in all-sample compared with amoxicillin–clavulanic acid-resistant isolates (31.8%, 0.8–96.3) (Table 1). Vm pooled resistance against amoxicillin–clavulanic acid was also high in seafoods with 27.2% (0.04–99.7). Ampicillin–sulbactam resistance was significantly different in seafoods (Figure S7).

### 2.13. Heterogeneity and Publication Bias

The between-study heterogeneity in the all-sample pooled Vm resistance prevalence was generally low to moderates (I^2^ ≤ 75) with an exception to amikacin (87.7%, 75.6; 93.8), gentamicin (78.6%, 63.1–87.6), kanamycin (75.4%, 52.6–87.2), cefuroxime (82.3%, 45.7–94.3), amoxicillin (78.9%, 49.7–91.1), ampicillin (80.7%, 70.7–87.2), sulfamethoxazole (79.5%, 58.1–90.0), and ampicillin–sulbactam (84.4%, 53.5–94.8) with I^2^ > 75% (Table 1). Also, there was generally no publication bias identified by LFKi in all cases. However, Egger’s regression test identified bias in the pooled ciprofloxacin-resistant (intercept = −2.64, *p* = 0.03), tetracycline-resistant (intercept = −4.10, *p* < 0.001), and trimethoprim–sulfamethoxazole-resistant (intercept = −3.75, 0.002) (Table 1) isolates.

### 2.14. World Bank Income; GNIpc = Gross National Income per Capita. Vm Antibiotic Resistance Burden

The sample-specific comparative Vm resistance burden in terms of ARRI is presented in Table 2. Seafood had the highest Vm resistance burden (ARRI = 50.84), followed by environmental waters (ARRI = 46.53), and the least in humans (ARRI = 0.01). In addition, Vm ARRI was higher in shellfish (10.97) compared to fish (2.08). The cumulative Vm-resistant instances (CVMRIs) varied from 2 (human) to 1179 (environmental water) and the total antibiotics tested varied from 5 (human) to 143 (seafood).

**Figure 1:**
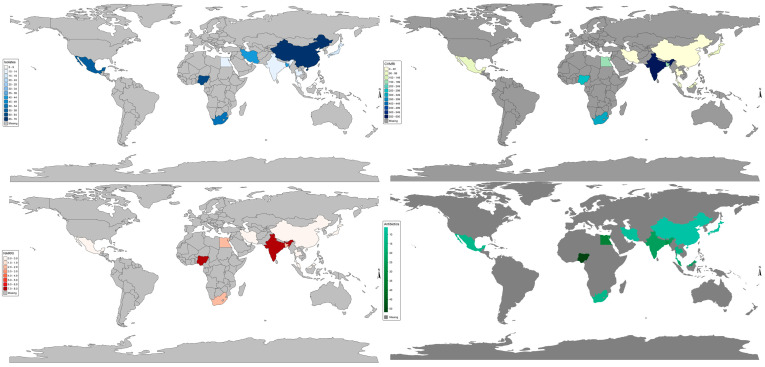
National antimicrobial resistance risk index burden (NARRI) of *V. mimicus*. CAVMRI = Sum of all *V. mimicus*-resistant instances for a nation.

Figure 1 presents nation-specific Vm resistance burden (ARRI). Vm ARRI was particularly higher in Nigeria (7.78) and India (7.35), followed by Egypt (2.84), Bangladesh (2.80), and South Africa (2.13) and <1 in Malaysia, Mexico, Japan, China, Iran, Brazil, and Thailand. CVMRI was the highest in India (575) and the least in Thailand (5).

**Figure 2:**
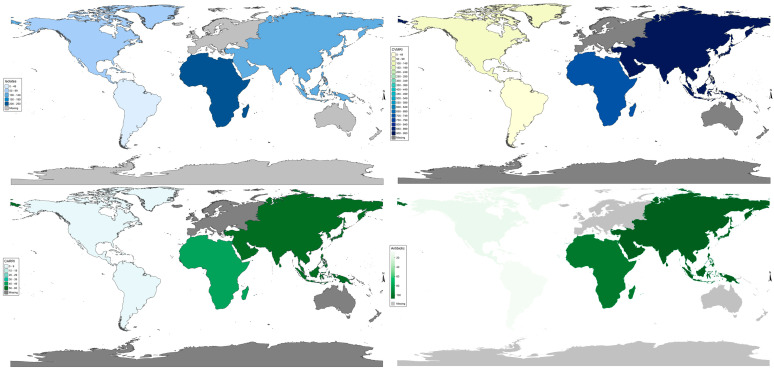
Continental antimicrobial resistance risk index burden (NARRI) of *V. mimicus*. CAVMRI = Sum of all *V. mimicus*−resistant instances for a continent.

Asia (56.91) and Africa (40.12) have high burden of Vm resistance compared to North America (0.59) and South America (0.06) (Figure 2).

## 3. Discussion

The present study examined the prevalence of various antimicrobial resistance in seafood-derived and aquatic environment-derived Vm isolates for the current state of knowledge and critical gaps for enhanced surveillance and research. The findings revealed low diversity of seafoods tested for Vm AMR and variability in the antibiotics tested. Monitoring of Va AMR should further be extended to other seafoods and essential *Vibrio*-specific antibiotics.

Vm isolates from seafoods and aquatic environments consistently show high susceptibility to aminoglycosides in this study except with above average resistance against streptomycin. This implies that resistance to aminoglycosides such as kanamycin, amikacin, and gentamicin among Vm is currently very low. A recent survey of Vm from fish, prawn, crab, and mussel samples in South Africa found 100% of isolates susceptible to amikacin and gentamicin [5]. Similarly, complete susceptibility to gentamicin and streptomycin were seen in environmental *Vibrio* from Indian coastal waters [34]. The heterogeneity between aminoglycoside resistance patterns of Vm by sources appears minimal for both seafood-derived and waterborne Vm and tends to remain sensitive to these agents. Clinically, this suggests that some aminoglycosides remain effective for treating severe Vm infections in humans or aquaculture animals. For seafood safety, the low resistance means contamination with Vm probably does not undermine amikacin-based or gentamicin-based therapies. However, a limitation of the current dataset is the small number of Vm isolates tested for every aminoglycoside; a larger number of Vm isolates or surveys across more regions would better picture global aminoglycoside resistance. As a mitigation strategy, continued prudent use of aminoglycosides in aquaculture and monitoring of resistance genes is advisable. Stewardship policies should discourage indiscriminate use of gentamicin/amikacin in fisheries, preserving their efficacy, and ensure effluents from farms are treated to prevent dissemination of any emergent aminoglycoside resistance into the broader environment.

Vm isolates generally remain highly susceptible to carbapenems (e.g., imipenem, meropenem) in this current study. For food safety and public health, this is reassuring as carbapenems are last-resort drugs for severe infections. Gxalo and collegues reported high susceptibility among freshwater Vm isolates to imipenem (52.5%) and meropenem (62.5%) [31]. There is little heterogeneity in carbapenem resistance patterns in both clinical and environmental Vm isolates but they typically show negligible carbapenem resistance. However, rising carbapenem use in human medicine and hospital effluents could eventually introduce resistance genes into aquatic Vm. Notably, some *Vibrio* have acquired OXA-type or metallo-β-lactamases, but at very low frequency [5,31]. The current dataset, being limited in sample size and geographic scope, likely underestimates the true global picture of carbapenem-resistant Vm. It would be valuable to screen more Vm isolates for carbapenem resistance and carbapenem bla genes (e.g., blaOXA, blaIMP) to detect any emerging trends. As a policy response, hospital wastewater effluents should be properly treated to remove carbapenem-resistant bacteria before release. Similarly, aquaculture should avoid use of broad-spectrum β-lactams (often combined with carbapenems in therapy) to reduce selective pressure. Maintaining carbapenems as a last resort requires one-health coordination between human and environmental sectors.

Cephalosporin resistance is appreciable in Vm in the current findings, especially for earlier-generation agents. Also, the data suggests heterogeneity by source is small, but high cephalosporin resistance appears broadly in both seafood-derived and waterborne Vm isolates. This concerns seafood safety because cephalosporins (e.g., cefotaxime, ceftazidime) are used in human medicine to treat Gram-negative infections. A previous freshwater Vm survey in South Africa, showed 82.5% resistance to cefuroxime (a second-generation cephalosporin) [31] showing agreement with the current result. Although data specifically on third-generation cephalosporins in Vm are sparse, the presence of ESBL genes (e.g., blaTEM, blaSHV) in many aquatic *Vibrio* hints at possible third-generation cephalosporin resistance as well [31]. Environmental contamination with resistant Vm-carrying ESBL could seed resistance into pathogens through mobile elements. Potential pathways include antibiotic runoff from aquaculture farms (where cephalosporins may be used) and faecal contamination carrying resistant *Vibrio*. The current pooled dataset may underestimate regional variability such as limited sampling and might miss hotspots where cephalosporin resistance is even higher. Also, the few Vm isolates tested against each cephalosporin could not provide a holistic overview of cephalosporin resistance in Vm. Improving representativeness of Vm isolates in terms of diverse locations and larger isolate numbers would clarify true cephalosporin resistance prevalence. As a policy measure, surveillance of ESBL-producing *Vibrio* and Vm in seafood is warranted. Regulatory agencies should restrict non-essential use of cephalosporins in aquaculture. Wastewater from fish farms and markets should also be disinfected to kill resistant strains. Promotion of good aquaculture practices (vaccination, probiotics) can reduce reliance on these antibiotics and slow the spread of cephalosporin resistance.

Vm chloramphenicol resistance was relatively low and moderate in this investigation. This suggests that chloramphenicols are still active against Vm. Abioye and colleagues found all Vm isolates to be chloramphenicol-sensitive [5]. Thus, chloramphenicol therapy could be effective in the event of seafood contamination, aquaculture, and human vibriosis. A crucial regulation safeguarding the effectiveness of chloramphenicol and its use in fish farming needs to be put in place.

Fluoroquinolones generally remain effective against Vm according to the current dataset, though some variability exists. This suggests that fluoroquinolones such as norfloxacin and ciprofloxacin (a primary drug for severe *Vibrio* infections) are largely still active against Vm. While previous studies have reported low ciprofloxacin resistance (0%; 16%) and norfloxacin resistance (0%) among *Vibrio* and Vm isolates [31,34], ofloxacin resistance was higher at 62% [31]. The fluoroquinolone resistance heterogeneity between isolates from different sources in the current study can be notable as environmental effluents might harbour strains with gyrA/parC mutations or plasmid-encoded qnr genes. Indeed, mutations in gyrA/B have been detected in Vm, indicating a capacity for fluoroquinolone resistance [5]. For public health, high susceptibility to ciprofloxacin is good, but any rise in resistance would compromise cholera-like illness treatments. Contamination of aquaculture water with fluoroquinolones (widely used in fish farming) can select for resistance. For example, studies have shown that intensive shrimp farming often uses enrofloxacin, norfloxacin, or oxolinic acid, leading to *Vibrio* isolates with lowered susceptibility [35]. The current data from seafood might under-represent regions where quinolones are heavily used. To mitigate spread, strict regulation of fluoroquinolone use in aquaculture is crucial. Environmental monitoring should include testing fish-farm water for fluoroquinolone residues and resistant *Vibrio*. Policies encouraging alternative practices (e.g., improved hygiene, vaccines) will reduce reliance on quinolones and help keep Vm fluoroquinolone-sensitive.

Macrolide resistance in Vm is high in the current investigation. This probably reflects Vm’s intrinsic tolerance or widespread acquisition of erm or efflux genes. Azithromycin and erythromycin resistance rates approach 100% in several studies of environmental Vm [31,36]. In freshwater isolates from India, all examined *Vibrio* (though not explicitly Vm only) were resistant to azithromycin [36]. Similarly, wild *Vibrio* from birds showed 88% erythromycin resistance [36]. Macrolide resistance heterogeneity by source seems minimal—whether from seafood or water, Vm generally carries macrolide resistance. The implication is that macrolides (used for *Vibrio* colitis or wound infections) may be ineffective; reliance on them poses a risk of treatment failure. For seafood safety, if Vm on raw fish is macrolide-resistant, then human infections from such seafood would not respond to erythromycin/azithromycin therapy. Macrolide resistance genes could spread via mobile elements into more pathogenic *Vibrio* species. Pathways include human sewage (macrolides are commonly prescribed) and use of related antibiotics in farm animals. The current dataset may not have explicitly pictured true macrolide resistance data for Vm by environment, which is a weakness, but the overall trend is clear. Policies should thus discourage empirical macrolide use for *Vibrio* and prompt susceptibility testing. Seafood producers should be educated that macrolides offer little protective advantage for Vm infections. Surveillance could focus on detecting ermB or mphA genes in aquatic settings. Finally, ensuring coliforms and *Vibrio* are controlled in fish-processing waters will reduce dissemination of macrolide-resistant bacteria into food.

Resistance to penicillins (especially ampicillin) is ubiquitous and high in Vm according to the current pooled dataset. This reflects the widespread presence of β-lactamases in Vm. In freshwater surveys, 95% Vm isolates were ampicillin-resistant [31] and penicillin G showed very high resistance up to 86% in bird-derived *Vibrio* [36]. The current dataset indicates Vm from seafood and water are similarly resistant, reflecting the widespread presence of β-lactamases. For seafood safety, this means raw Vm contamination could not be controlled by simple penicillin treatments. For human health, it means empiric therapy with ampicillin for suspected *Vibrio* infection is likely to fail unless combined with inhibitors (and even that is often insufficient). These high penicillin resistances are driven by horizontal gene transfer of blaTEM, blaOXA, etc. [31]. Contamination routes include effluents from animal farms (where penicillins are heavily used) and discharges from healthcare settings. The weaknesses of the current pooled dataset are small number of Vm tested and geographic limitation—resistance genes vary by region and such, it could not provide holistic global penicillin-resistant Vm, so additional surveillance in different seafoods and environments is needed. Policy implications include phasing out non-therapeutic penicillin use in aquaculture and enhancing wastewater treatment to degrade β-lactamase genes. Where farmed seafood is processed, monitoring for β-lactam-resistant *Vibrio* could be instituted. Education campaigns can inform farmers that penicillins do not reliably prevent vibriosis and encourage vaccines or bacteriophage as alternatives in aquaculture.

Polymyxin (colistin) resistance in Vm is high in the current findings. The reasons likely lie in species-specific traits; while *V. cholerae* and related vibrios are intrinsically polymyxin-resistant, other species may not be [37]. Thus, colistin resistance heterogeneity is variable and context-dependent. In one study, 100% Vm freshwater isolates were resistant to polymyxin B [31], whereas in an environmental survey, all *Vibrio* were sensitive to polymyxin B [34]. For seafood safety, polymyxin is rarely used in aquaculture (due to toxicity) but is a last-resort human drug for multi-drug-resistant infections. The presence of polymyxin-resistant Vm in coastal waters or seafoods is a warning, since mcr genes have been found on *Vibrio* plasmids [5]. Spread pathways include runoff from farms where colistin is still used (in some countries, though many have banned it), and transfer of mcr from Enterobacteriaceae in manure [38]. The current pooled dataset on polymyxin-resistant Vm is sparse and limited in geography to reflect the true global polymyxin-resistant Vm; more systematic screening for mcr-1/mcr-3 in aquatic *Vibrio* is needed. Given the public health risk, authorities should enforce bans on colistin in livestock and aquaculture globally. Environmental monitoring of coastal waters and farm effluents should include colistin residue and mcr gene surveillance. In the meantime, avoid relying on polymyxin to clear *Vibrio* contamination in seafood—instead, use cooking or other physical decontamination methods.

The current dataset suggests moderate sulfonamide resistance prevalence in Vm. Sulfonamide resistance genes (e.g., sul1, sul2) are frequently found in aquatic *Vibrio*. In *Vibrio* isolates in one study, 35% were co-trimoxazole-resistant [34], and 7% carried sul1 in another study [5]. Vm specifically often remains somewhat sensitive, but a substantial minority are resistant to sulfamethoxazole/trimethoprim. Sulfonamide resistance heterogeneity exists. sul1 carriage was 7% overall in one study, implying some water isolates are carrying class 1 integrons [5]. For seafood safety, sulfonamide resistance in Vm means these antibiotics (often used for mild infections) may not clear contamination. In the community, widespread use of trimethoprim–sulfamethoxazole selects for these genes. Aquaculture use of sulfonamides (common in shrimp farming) also drives resistance. Pathways for spread include horizontal transfer via integrons in polluted waters. A weakness of the current sulfonamide-resistant Vm dataset is its sparsity and geographic limitation. To mitigate this, regulations should restrict prophylactic use of sulfonamides in fish farms. Outbreaks traced to seawater contamination should consider testing for sul genes. Where irrigation with wastewater occurs, measures to remove antibiotics would help reduce selective pressure in estuarine environments.

Tetracycline resistance in Vm appears relatively low compared to oxytetracycline and doxycycline in the current study. This suggests that many Vm still can be controlled by tetracycline therapy. In environmental surveys, *Vibrio* spp. often remain sensitive to tetracycline [34] and show 0% resistance to oxytetracycline in aquatic isolates [36]. However, tetracyclines are also heavily used in aquaculture worldwide, and resistance can emerge. For human health, tetracyclines (e.g., doxycycline) are frontline *Vibrio* drugs, especially for *V. cholerae* and *V. vulnificus* [39]. Low resistance means these drugs remain effective, but vigilance is needed as aquaculture use could select resistant clones. Aquaculture pathways involve direct administration of oxytetracycline in feed. Effluent from aquaculture may carry tetracycline residues and select for tet-bearing bacteria in surrounding waters. Nonetheless, to slow future resistance rise, some policies already limit tetracycline use or require withdrawal periods. Recommendations include encouraging vaccine use in farmed shrimp/fish to reduce antibiotic need, and monitoring tet gene prevalence in coastal waters near farms. If resistance begins to increase, guidelines might shift to emphasise other antibiotic classes or non-antibiotic interventions.

Vm was sensitive to trimethoprim–sulfamethoxazole (TMP-SMX) in the current dataset. This suggests that many Vm still can be controlled by TMP-SMX therapy. However, Abioye and colleagues found 22.2% Vm isolates to resistant against TMP-SMX [5]. Also, in another report, 35% of *Vibrio* isolates were TMP-SMX resistant [34]. TMP-SMX resistance heterogeneity depends on presence of dfr genes [5] and TMP-SMX combination should be used with caution. For seafood safety, failure of TMP-SMX means gastroenteritis from contaminated seafood may require other drugs. Environmental contamination (e.g., hospital sewage) introducing dfr genes into water could further raise resistance. The current dataset likely cannot show the true trend of TMP-SMX resistance in Vm due to regional limitation and sparsity. Policy actions include continuing guidelines that use TMP-SMX only when susceptibility is confirmed and treating sewage to remove antibiotics. Aquaculture should avoid blanket use of sulfa drugs, favouring targeted disease management.

Vm showed moderate resistance against β-Lactam/β-Lactamase inhibitor combinations like amoxicillin–clavulanate intended to overcome β-lactamases. This implies that such combinations are not reliably effective. Vm often carries broad-spectrum β-lactamases that resist even these combinations. Freshwater Vm have shown high resistance (72.5%) to ampicillin–sulbactam [31]. From a food safety perspective, reliance on these inhibitor combos to safeguard seafood is not warranted. Clinically, this class is losing value against aquatic *Vibrio*. The spread of ESBL or AmpC enzymes via plasmids means that even newer inhibitors (e.g., clavulanate) are bypassed. A weakness of existing data is few studies explicitly test piperacillin/tazobactam or newer inhibitors in *Vibrio*. As a precaution, authorities should restrict the use of penicillin/inhibitor combinations in aquaculture (they are often in the same priority class as other penicillins). Surveillance to detect blaCTX-M or blaOXA genes in environmental *Vibrio* could inform when these drugs are no longer advisable in human therapy. In the meantime, emphasis should be on preventing infection (hygienic seafood handling) rather than relying on high-end antibiotics.

Overall, the pattern of resistance in Vm from seafood and water in the current study shows multidrug resistance concerns: high rates against penicillins, cephalosporins, macrolides and sometimes polymyxins, with lower resistance to aminoglycosides, fluoroquinolones, and tetracyclines. This is corroborated by previous studies [5,31,34]. This heterogeneity underscores the impact of antibiotic use patterns in different environments. For seafood safety, it means that raw or undercooked seafood could harbour Vm that are resistant to many antibiotics, necessitating careful cooking and possibly monitoring of resistance in seafood supply chains. For environmental health, the data reflects contamination of waters by antibiotic residues and resistant bacteria from farms, hospitals, and communities. Public health implications include risk of hard-to-treat vibriosis if these strains infect humans—which is particularly concerning as Vm can cause cholera-like diarrhoea [5].

The current data reveal that antibiotic resistance burdens of Vm in seafood (ARRI ≈ 50) and waters (ARRI ≈ 46) exceeded that of human isolates (ARRI ≈ 0.01) greatly. Also, it identified Asia/Africa as hotspots of Vm antibiotic resistance burden. Generally, Asia/Africa AMR hotspots/burden are driven by weak regulation and implementations. Tighter AMR and Vm monitoring in aquaculture, shellfish, seafood, and reused wastewater is critically needed to protect public health especially in Asia/Africa. Abioye and co-workers reported 48% *Vibrio* from seafood with a high-risk Multiple Antibiotic Risk Index (MARI > 0.2) [5]. Generally, ARRI allows cross-group Vm resistance burden comparability; it is susceptible to sample size/antibiotic panel’s inequalities. On a worthy note, ARRI underlined the complications of AMR risks (AMRRs) in the like manner of risk quotient, MARI, comparative AMRR index, and other risk indices [40].

Key pathways for resistance spread likely include the following: (1) aquaculture practices, where prophylactic or therapeutic antibiotics in fish/shrimp feed select resistant Vm, which then enter waters; (2) waterborne dissemination, such as release of untreated effluent from markets, farms or sewage introducing resistant genes into coastal waters; and (3) food chain transfer, where contaminated seafood passes resistant vibrios to consumers. The current dataset has limitations including limited geographic coverage and a small number of Vm isolates tested against the antibiotics.

Considering these findings, policy recommendations include enforcing stricter regulations on antibiotic use in aquaculture (banning critical drug classes, veterinary prescription requirements); improving wastewater treatment to inactivate antibiotics and resistant bacteria; implementing one-health surveillance linking environmental, animal and human health data; and promoting education of stakeholders (farmers, traders, consumers) about AMR risks. Where possible, encouraging alternatives to antibiotics (e.g., vaccines, probiotics, phage therapy) and ensuring proper cooking of seafood will mitigate the impact of resistant Vm. These evidence-based strategies will help preserve the efficacy of current antibiotics and protect public health.

## 4. Materials and Methods

### 4.1. Study Design and Data Strategy

The research design involved a systematic search of multiple databases to collect primary data on Vm AMR. Published articles containing primary data on the antimicrobial susceptibility of Vm isolates from seafoods and aquatic environment were sourced from EBSCOhost, Scopus, WoS, and PubMed from the inception till 10 May 2025 without imposing any specific restrictions. The generalised topical search query used in the databases was “(mimicus AND (resistan* OR nonsuscepti* OR antibiogram* OR antibiotic* OR antimicrobial*))”. The specific details of the databases searched are presented in the Supplementary Material. The study was approved by the College of Agriculture and Environmental Sciences_Health REC, University of South Africa with the ethical clearance reference number 2025/CAES_HREC/6998.

### 4.2. Eligibility Criteria

Studies (articles) that performed antimicrobial assays on Vm from any sample type were included using PRISMA protocols. The study must be available in full text and detail the antimicrobial assay tested on Vm isolates. The source (sample) and number of Vm isolates tested must be documented. The antibiotics tested and antimicrobial susceptibility test approach (e.g., microdilution, disc diffusion test, E-test, etc.) must be recorded.

### 4.3. Data Management

The Vm data retrieved from the databases were merged in Zotero (version 7.0.13). After duplicate merging, the collection was exported into Excel version 2016 for screening of titles and abstracts. The full texts of the eligible studies were retrieved, read, and the targeted research data were collated into predesigned forms in two trials. Data from the trials were validated when the union set and intersection set of the two trials are equal and by the co-authors. Disagreements were harmonised by discussion. The entire workflow is summarised in Figure S1.

### 4.4. Data Items and Treatment

The data collected include author, year, number of Vm isolates tested, number of resistant Vm isolates for each tested antibiotic, antimicrobial susceptibility testing method, sample type, and country of origin. The quality of individual primary data was assessed based on Vm confirmation and the description of antimicrobial susceptibility testing (AST) methods.

### 4.5. Data Synthesis

The data collected were grouped by sample type (seafoods, environmental water, and clinical sample), logit transformed [41] and fitted to a random intercept logistic regression model [42]. Heterogeneity in the data was assessed using the maximum likelihood estimation [43]. Publication bias was evaluated using Egger’s regression [44] when the number of the ungrouped studies pooled was ≤10; otherwise, the Luis Furuya-Kanamori index (LFKi) was applied [45]. Furthermore, a sample-based mixed-effects subgroup analysis was conducted to estimate sample-specific prevalence [46].

Furthermore, Vm antibiotic resistance burden was assessed using antimicrobial resistance risk index (ARRI) metrics for cross-class and cross-territorial comparisons using Equation (1) [47]. ARRI is the total Vm-resistant instances to all antibiotics tested for a specific sample (*s*) or nation (*n*) or continent (*c*) ($\sum_{i=1}^{j}Rt_{s,n,c,P}$) divided by the overall Vm-resistant instances for all samples (*s_s_*) or nations (*n_s_*), or continents (*c_s_*) ($\sum_{n=1}^{k}Rt_{s_{s}\left|n_{s}\right|c_{s},Vm}$) multiplied by the number of antibiotics used/tested for/by the sample/nation/continent to which Vm was resistant to ($\sum_{t=1}^{s}RtAc_{s,n,c,Vm}$).

(1) $$ARRI_{s\left|n\right|c}=\left(\frac{\sum_{i=1}^{j}Rt_{s\left|n\right|c,Vm}}{\sum_{n=1}^{k}Rt_{s_{s}\left|n_{s}\right|c_{s},Vm}}\right)\times \sum_{t=1}^{s}RtAc_{s\left|n\right|c,Vm}\ldots ,$$

where *j*, *k*, and *s* = maximum number of instances in each case. Data was grouped into environmental water, seafood (shellfish, fish), and human samples for sample-specific ARRI computation. All analyses were performed using R v.4.4.3 (2025-02-28 ucrt).

## Figures and Tables

**Table 1:** Pooled sample and sample-specific prevalence of different antibiotic resistance in 423 *V. mimicus* isolates.

				All Samples				Seafood			Environmental Water	
s/n	Antibiotic	Class	Pooled study	Prevalence (95% CI)	I^2^ (95%CI)	ET/LFKi	k	Prevalence (95% CI)	I^2^	k	Prevalence (95% CI)	I^2^
1	Amikacin	Aminoglycosides	k = 6; e = 54	22.6 (4.6–64.1)	87.7 (75.6; 93.8)	0	4	19.2 (2.0–73.7)	70.6	2	30.8 (0.0–100.0)	89.1
2	Gentamicin	Aminoglycosides	k = 12; e = 53	16.5 (2.8–57.4)	78.6 (63.1–87.6)	0.57 (−2.72–3.85, 0.74)	9	26.7 (3.7–77.6)	80.1	3	5.2 (0.1–80.4)	0
3	Kanamycin	Aminoglycosides	k = 9; e = 76	28.8 (3.5–81.9)	75.4 (52.6–87.2)	0	4	50.3 (5.8–94.4)	0	4	25.1 (0.2–98.6)	89.9
4	Streptomycin	Aminoglycosides	k = 9; e = 71	54.6 (15.8–88.5)	67.8 (35.2–84.0)	0	5	51.8 (9.5–91.7)	49.9	3	79.3 (0.1–100.0)	81.4
5	Imipenem	Carbapenems	k = 6; e = 24	10.7 (2.5–35.9)	31.3 (0.0–72.1)	0	3	2.1 (0.0–99.9)	0	3	16.3 (1.9–65.6)	71.7
6	Meropenem	Carbapenems	k = 5; e = 27	12.3 (1.6–54.6)	0.0 (0.0–79.2)	0	2	6.6 (0.0–100.0)	0	3	16.9 (1.7–70.6)	0
7	Cefotaxime	Cephalosporins	k = 7; e = 30	8.9 (2.3–29.3)	70.0 (34.3–86.3)	0	5	7.3 (2.3–21.13)	11.3	2	8.6 (0.0–100.0)	0
8	Cefuroxime	Cephalosporins	k = 3; e = 77	60.1 (2.3–99.0)	82.3 (45.7–94.3)	0	1	10.0 (1.4–46.7)	–	2	80.9 (13.1–99.2)	0
9	Cephalothin	Cephalosporins	k = 5; e = 30	28.7 (13.3–51.4)	62.9 (2.0–86.0)	0	4	27.6 (9.3–58.5)	70.1	1	35.7 (15.7–62.4)	–
10	Chloramphenicol	Chloramphenicol	k = 10; e = 100	32.3 (2.7–89.0)	68.8 (39.8–83.8)	−1.14 (−4.02–1.73, 0.5)	6	20.8 (1.1–85.9)	64.9	4	51.9 (0.0–100.0)	0
11	Ciprofloxacin	Fluoroquinolones and Quinolones	k = 10; e = 49	9.9 (1.7–41.6)	71.0 (44.7–84.8)	−2.64 (−4.66–−0.62, 0.03)	7	8.6 (0.7–56.4)	34.5	3	12.9 (0.1–97.3)	89.3
12	Norfloxacin	Fluoroquinolones and Quinolones	k = 8; e = 56	8.9 (0.4–70.9)	20.8 (0.0–63.1)	0	5	18.0 (1.1–81.5)	0	3	1.4 (0.0–100.0)	0
13	Nalidixic acid	Fluoroquinolones and Quinolones	k = 5; e = 50	47.2 (17.3–79.4)	0.0 (0.0–79.2)	0	4	39.7 (5.2–88.7)	0	1	59.3 (45.8–71.5)	–
14	Ofloxacin	Fluoroquinolones and Quinolones	k = 4; e = 70	34.0 (1.7–94.0)	69.1 (10.8–89.3)	0	2	6.6 (0.0–100.0)	0	2	71.0 (3.1–99.5)	76.2
15	Azithromycin	Macrolides and Azalides	k = 4; e = 50	65.8 (0.9–99.5)	72.2 (21.3–90.2)	0	2	46.2 (0.1–99.9)	44	2	94.3 (0.0–100.0)	0
16	Erythromycin	Macrolides and Azalides	k = 7; e = 29	21.5 (3.0–70.8)	51.7 (0.0–79.5)	0	6	17.2 (4.3–49.2)	0	1	92.9 (63.0–99.0)	–
17	Amoxicillin	Penicillins	k = 5; e = 46	83.7 (5.3–99.8)	78.9 (49.7–91.1)	0	3	98.0 (0.1–100.0)	0	2	21.0 (0.0–100.0)	91.3
18	Ampicillin	Penicillins	k = 19; e = 203	61.11 (24.5–88.4)	80.7 (70.7–87.2)	1.14; −1.26–3.53; 0.4	12	76.1 (17.2–98.0)	55.8	6	58.0 (19.4–88.8)	91.6
19	Penicillin	Penicillins	k = 4; e = 24	72.7 (43.5–90.3)	16.5 (0.0–87.2)	0	4	72.7 (43.5–90.3)	16.5	–	–	–
20	Colistin sulphate	Polymyxins	k = 3; e = 47	80.2 (0.0–100.0)	54.9 (0.0–87.1)	0	–	–	–	–	–	–
21	Sulfamethoxazole	Sulfonamides	k = 7; e = 40	38.3 (3.6–91.2)	79.5 (58.1–90.0)	0	6	25.8 (1.8–86.9)	71.6	1	92.9 (63.0–99.0)	–
22	Doxycycline	Tetracyclines	k = 5; e = 62	59.4 (3.6–98.3)	49.9 (0.0–81.6)	0	3	36.5 (0.0–100.0)	0	2	76.5 (7.9–99.2)	0
23	Tetracycline	Tetracyclines	k = 16; e = 102	13.5 (2.9–45.2)	69.7 (49.5–81.9)	−4.10 (−5.86–−2.34, <0.00)	10	12.8 (2.6–45.0)	52.5	5	35.1 (1.2–96.0)	77.4
24	Oxytetracycline	Tetracyclines	k = 4; e = 23	47.9 (26.1–70.5)	30.8 (0.0–75.0)	0	3	52.9 (20.3–83.3)	36.9	1	35.7 (15.7–62.4)	0
25	Trimethoprim–Sulfamethoxazole	Trimethoprim and Combinations	k = 12; e = 69	5.8 (0.7–36.1)	73.3 (52.5–85.0)	−3.75 (−5.48–−2.01, 0.002)	6	4.9 (0.4–41.0)	0	5	19.0 (0.4–92.6)	84.4
26	Amoxicillin–Clavulanic acid	β-Lactam/β-Lactamase Inhibitor Combos	k = 5; e = 46	31.8 (0.8–96.3)	47.3 (0.0–80.7)	0	4	27.2 (0.04–99.7)	0	1	48.2 (35.3–61.3)	–
27	Ampicillin–Sulbactam	β-Lactam/β-Lactamase Inhibitor Combos	k = 3; e = 36	43.3 (5.1–91.5)	84.4 (53.5–94.8)	0	2	25.9 (0.1–98.9)	0	1	72.5 (56.8–84.1)	–

k = number of studies pooled; e = observations; ET/LFKi = Egger’s regression test/Luis Furuya-Kanamori index.

**Table 2:** Sample-specific antibiotic resistance risk index of *V. mimicus*.

Sample	Cvmrni	Total Antibiotic Tested	ARRI
Seafoods	618	148	50.84
Environmental water	1179	71	46.53
Human	2	5	0.01
Shellfish	282	70	10.97
Fish	163	23	2.08

ARRI = antimicrobial resistance risk index; Cvmrni: cumulative V. mimicus resistance instance.

## Data Availability

All data generated or analysed in the current study are contained in the article and its Supplementary Materials.

## Supplementary Materials

The following supporting information can be downloaded at https://www.mdpi.com/article/10.3390/antibiotics14111075/s1, Figure S1. Flow diagram for selecting studies on antibiotic resistance in *Vibrio mimicus*; Figure S2. Prevalence of aminoglycoside antibiotics resistance in seafoods and environmental water *V. mimicus* isolates; Figure S3. Prevalence of carbapenems, macrolides, and azalides antibiotic resistance in seafoods and environmental water *V. mimicus* isolates; Figure S4. Prevalence of cephalosporins and chloramphenicol antibiotic resistance in seafoods and environmental water *V. mimicus* isolates; Figure S5. Prevalence of fluoro/quinolones antibiotic resistance in seafoods and environmental water *V. mimicus* isolates; Figure S6. Prevalence of penicillin antibiotic resistance in seafoods and environmental water V. mimicus isolates; Figure S7. Prevalence of sulfonamides and β-Lactam/β-Lactamase inhibitor antibiotic resistance in seafoods and environmental water *V. mimicus* isolates; Figure S8. Prevalence of tetracyclines and trimethoprim–sulfamethoxazole antibiotic resistance in seafoods and environmental water *V. mimicus* isolates. Table S1a. *V. mimicus* data; Table S1b. mv data from fewer than three studies.
