# Supplementary material for: Occurrence and Antibiotic Resistance Risk Burden of Vibrio mimicus Isolates from Seafood and Aquatic Environments
Source: Antibiotics (Basel). 2025 Oct 26;14(11):1075. doi: 10.3390/antibiotics14111075 (PMC12649710; doi:10.3390/antibiotics14111075)
Supplement: Supplementary file 1 [file antibiotics-14-01075-s001.zip › Figure S1_and_vm_supplementary_Results.pdf]

Supplementary methods

Search strategy

The search matrix involved:

PubMed

mimicus[tiab] & (resistan*[tiab] | nonsuscepti*[tiab] | antibiogram[tiab] | antibiotic[tiab] | antimicrobial*[tiab]) NOT (review[pt] | systematic review[pt] | meta-analysis[pt] | booksdocs[filter] | Retracted Publication[pt])

Scopus

Your query : (TITLE-ABS-KEY(mimicus AND (resistan* OR nonsuscepti* OR antibiogram* OR antibiotic* OR antimicrobial*)) AND (LIMIT-TO (DOCTYPE,"ar")))

Web of science:

TS=(mimicus AND (resistan* OR nonsuscepti* OR antibiogram* OR antibiotic* OR antimicrobial*)) AND DT=(Article) Timespan: All years (Publication Date)
Date Run: Sat May10 2025 17:21:17 GMT+0200 (South Africa Standard Time)

EBSCOhost

Query	Limiters/Expanders	Last Run Via
(((TI mimicus) AND (TI resistan* OR TI nonsuscepti* OR TI antibiogram* OR TI antibiotic* OR TI antimicrobial*)) OR ((TI mimicus) AND (AB resistan* OR AB nonsuscepti* OR AB antibiogram* OR AB antibiotic* OR AB antimicrobial*)) OR ((AB mimicus) AND (TI resistan* OR TI nonsuscepti* OR TI antibiogram* OR TI antibiotic* OR TI antimicrobial*)))	Limiters - Peer Reviewed; Publication Type: Academic Journal; Document Type: Article; Publication Type: Article; Document Type: Article; Document Type: Journal Article; Document Type: Journal Article; Test Type: Original; Publication Type: Article; Publication Type: Academic Journal; Document Type: Article; Publication Type: Academic Journal; Research Article; Publication Type: Journal Article; Publication Type: Journal Article; Publication Type: Academic Journal; Document Type: Article; Publication Type: Academic Journal; Document Type: Article; Publication Type: academic journal; Document Type: article; Publication Type: Academic Journal; Publication Type: Academic Journal;	Interface - EBSCOhost Research Databases<br>Search Screen - Advanced Search, Database - Academic Search Ultimate;Africa-Wide Information;APA PsycArticles;APA PsycBooks;APA PsycExtra;APA PsycInfo;APA PsycTests;Atla Religion Database with AtlaSerials;Business Source Ultimate;CAB Abstracts with Full Text;CINAHL Plus with Full Text;Core Curriculum;eBook Collection (EBSCOhost);eBook Open Access (OA) Collection (EBSCOhost);EconLit with Full Text;Education Source;Environment Complete;ERIC;Gender Studies Database;Global Health;GreenFILE;HBS Select Case Study Collection (eBook Sub) (EBSCOhost);Health Source Consumer Edition;Health Source: Nursing/Academic Edition;Hospitality & Tourism Complete;Humanities & Social Sciences Index Retrospective: 1907-1984 (H.W. Wilson);Index to Legal Periodicals Retrospective: 1908-1981 (H.W. Wilson);Inspec Archive - Science Abstracts 1898-1968;LGBTQ+ Source;Library & Information Science Source;Library, Information Science & Technology Abstracts;MasterFILE Premier;MasterFILE Premier Reference eBook Subscription

	Document Type: Article; Publication Type:<br>Academic Journal;<br>Document Type: Article; Publication Type:<br>Academic Journal;<br>Document Type: Article; Publication Type:<br>Academic Journal;<br>Document Type: Article; Publication Type:<br>Journal Paper;<br>Document Type: Article; Publication Type: Academic Journal; Publication Type:<br>Academic Journal;<br>Document Type: Article; Publication Type:<br>Academic Journal;<br>Document Type: Article; Publication Type:<br>Journal; Publication Type: Journal Article; Publication Type:<br>Article; Document Type: Article; Publication<br>Type: Article; Document<br>Type: Article;<br>Publication Type:<br>Article; Document Type: Article; Publication<br>Type: academic journal;<br>Document Type: article Expanders - Apply equivalent subjects<br>Search modes Proximity	(EBSCOhost);MEDLINE;Mental Measurements Yearbook with Tests in Print;MLA Directory of Periodicals;MLA International Bibliography;New Testament Abstracts;Newspaper Source;Old Testament Abstracts;OpenDissertations;Regional Business News;RILM Abstracts of Music Literature with Full Text;Teacher Reference Center;Waters & Oceans Worldwide;Wildlife & Ecology Studies Worldwide

Supplementary results

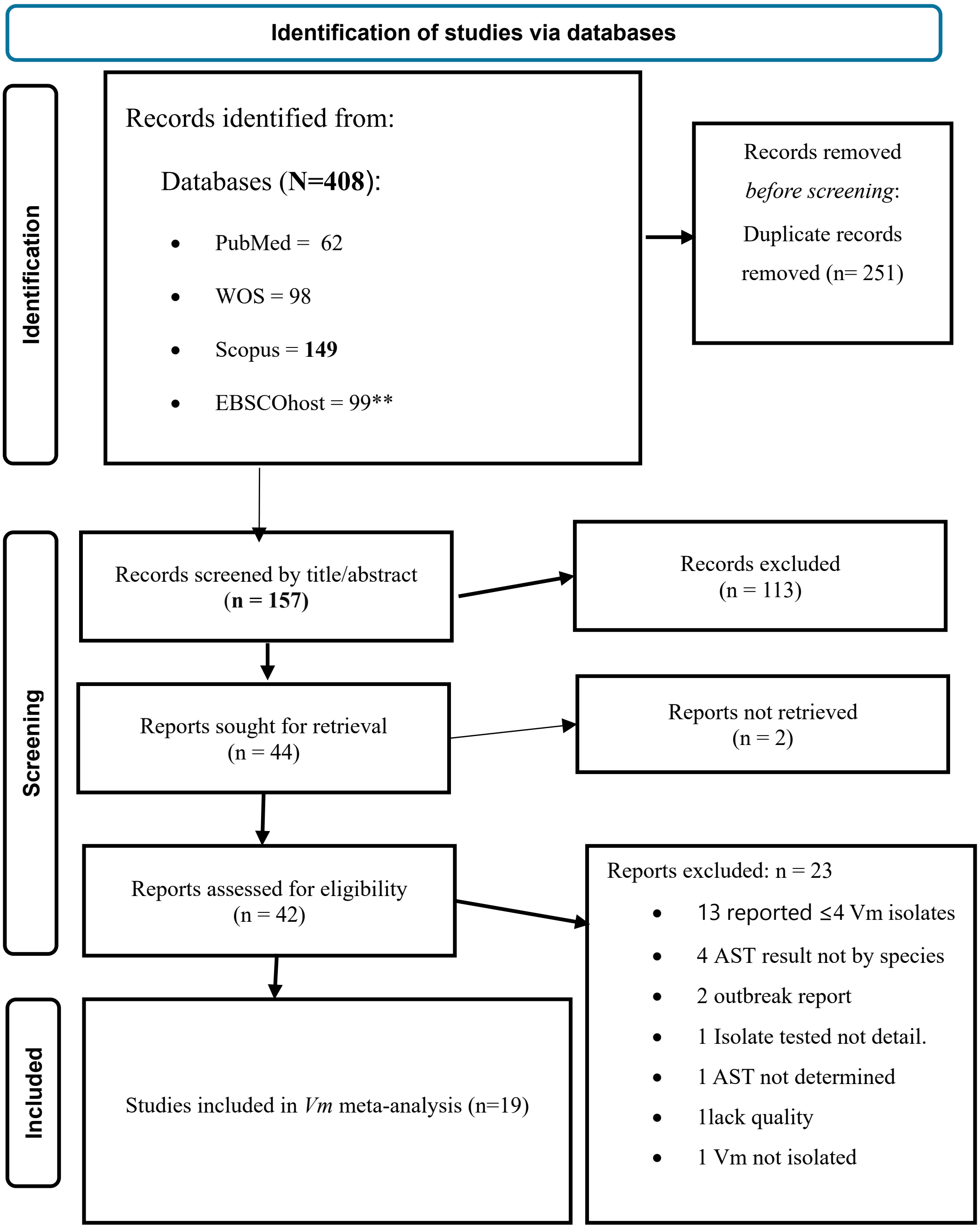

Figure S1. Flow diagram for selecting studies on antibiotic resistance in *Vibrio mimicus*.

**** last accessed on 11/12/2024, 11:22 AM**
