# Supplementary figures and images for: Occurrence and Antibiotic Resistance Risk Burden of Vibrio mimicus Isolates from Seafood and Aquatic Environments
Source: Antibiotics (Basel). 2025 Oct 26;14(11):1075. doi: 10.3390/antibiotics14111075 (PMC12649710; doi:10.3390/antibiotics14111075)

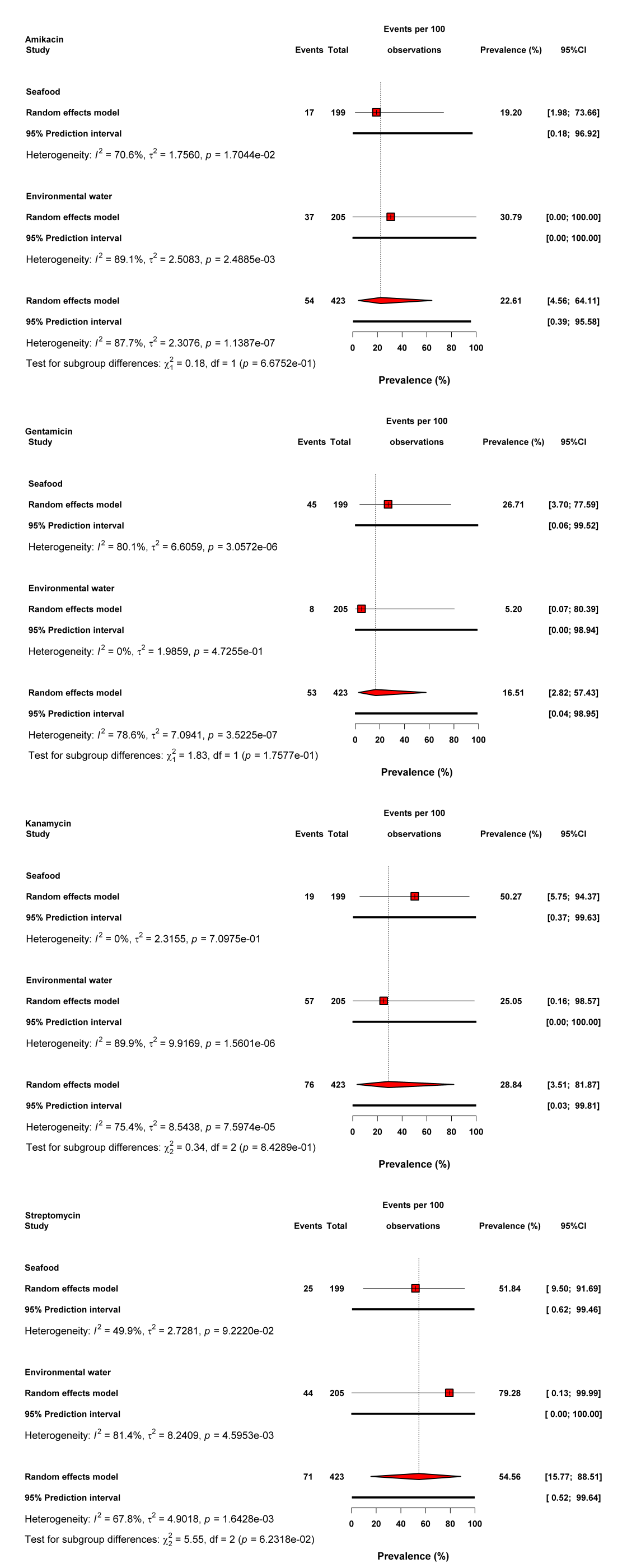

Supplement: Supplementary file 1 [file antibiotics-14-01075-s001.zip › FigureS2_aminoglycosides.tiff]

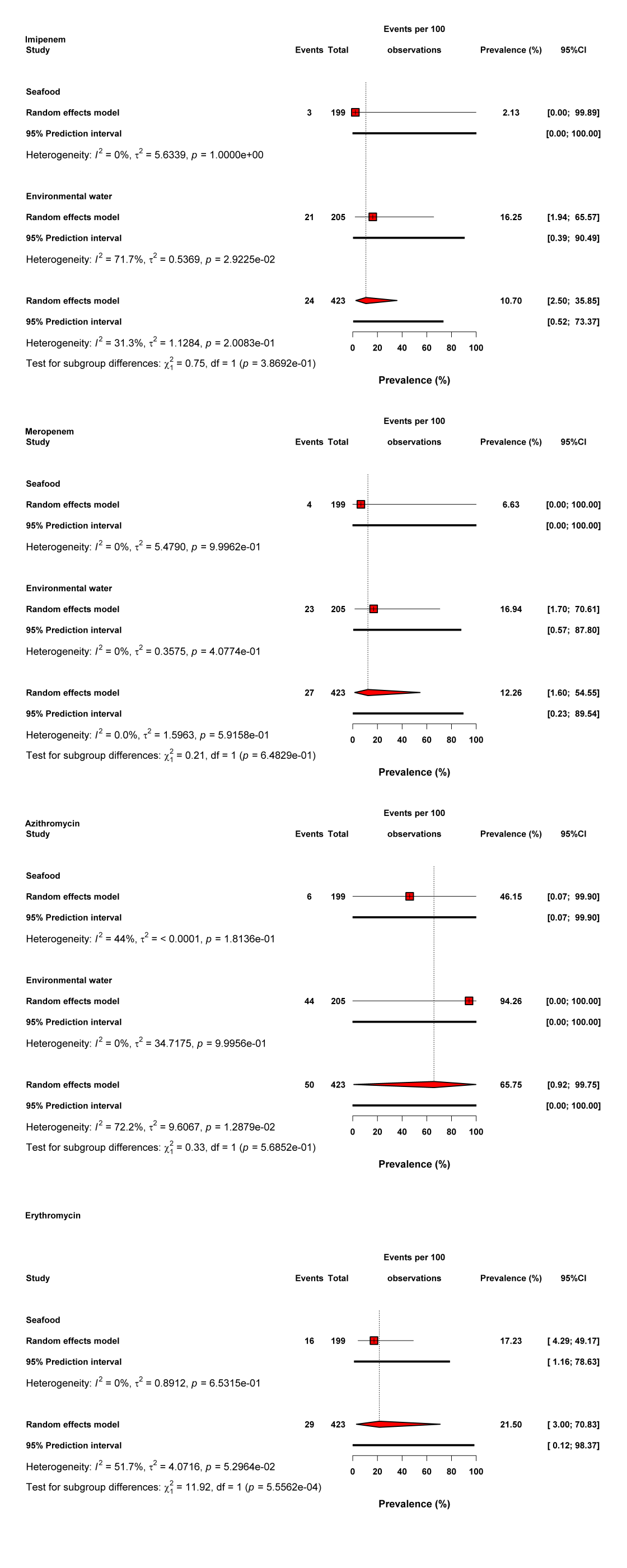

Supplement: Supplementary file 1 [file antibiotics-14-01075-s001.zip › FigureS3_Carbapenems_Macrolides-Azalides.tiff]

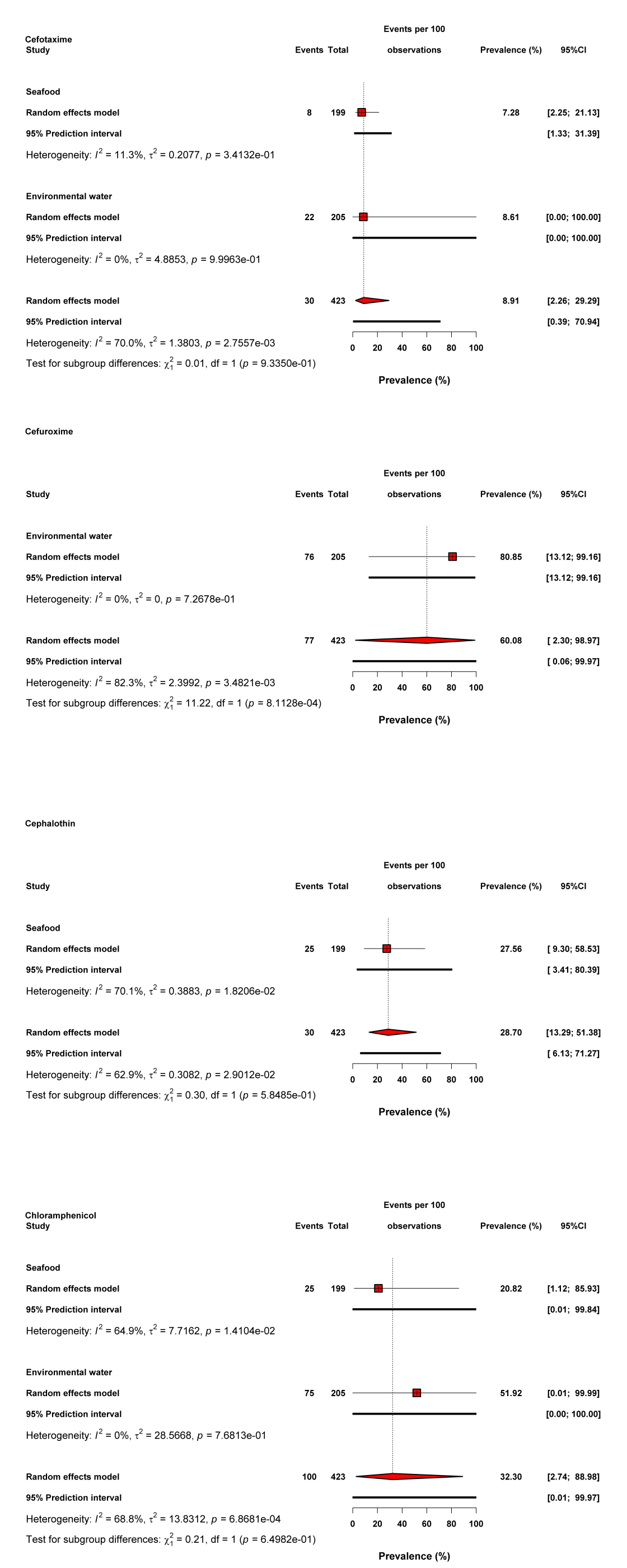

Supplement: Supplementary file 1 [file antibiotics-14-01075-s001.zip › FigureS4_Cephalosporins_chloramphenicol.tiff]

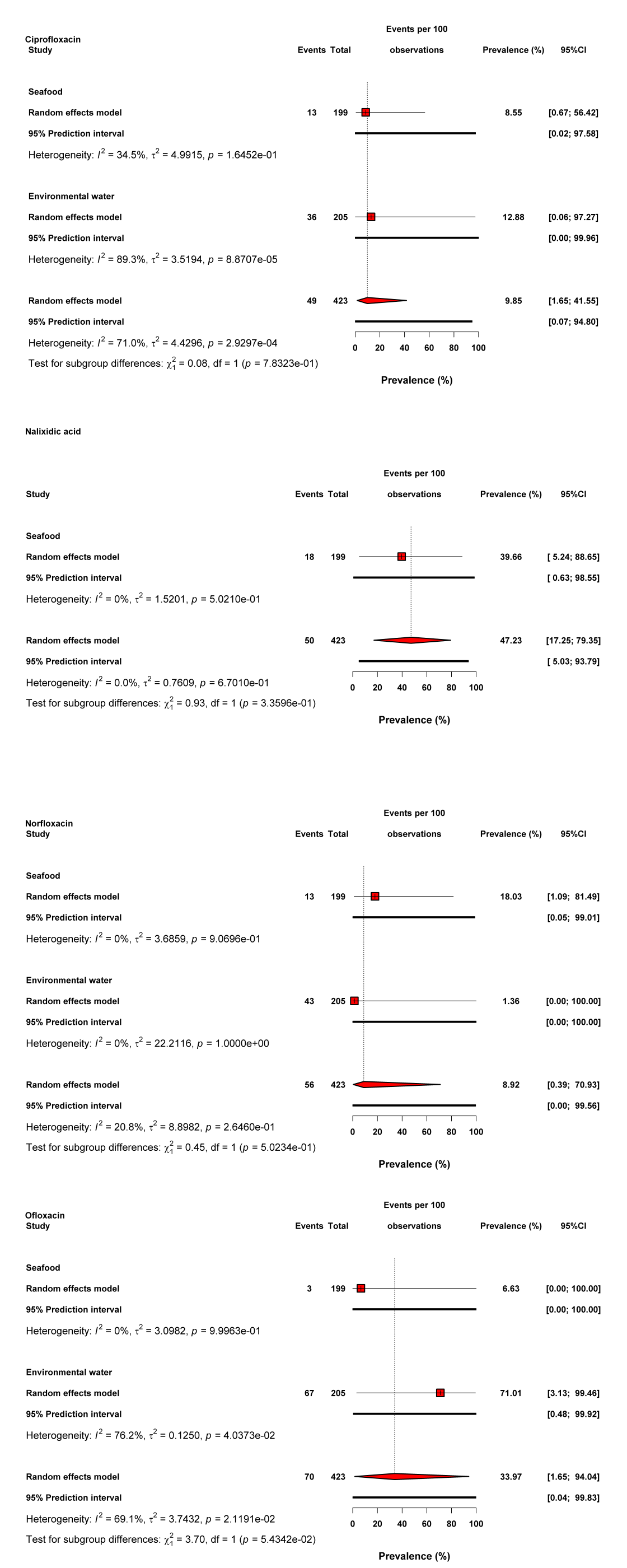

Supplement: Supplementary file 1 [file antibiotics-14-01075-s001.zip › FigureS5_Fluoroquinolones-Quinolones.tiff]

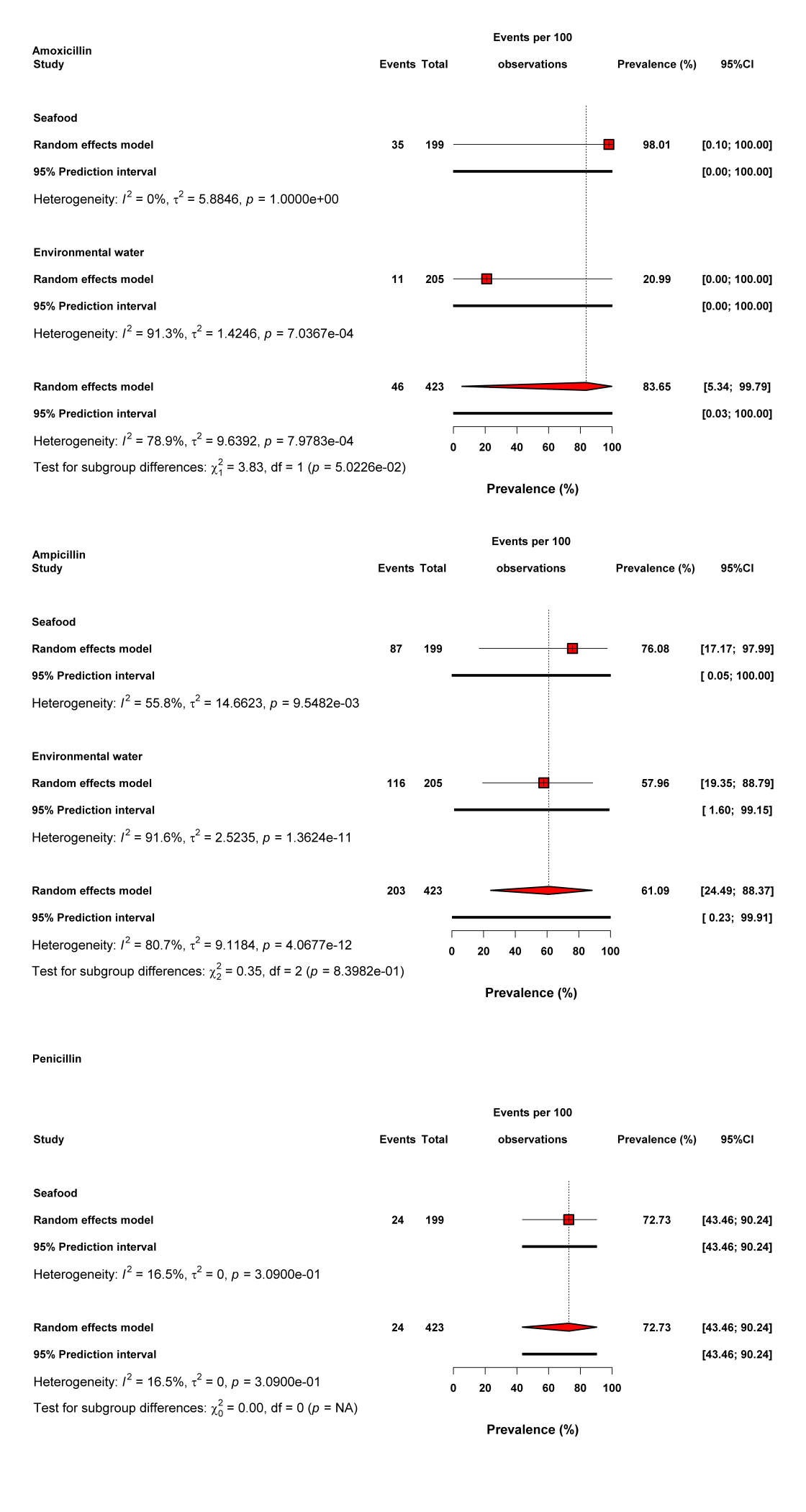

Supplement: Supplementary file 1 [file antibiotics-14-01075-s001.zip › FigureS6_Penicillins.tiff]

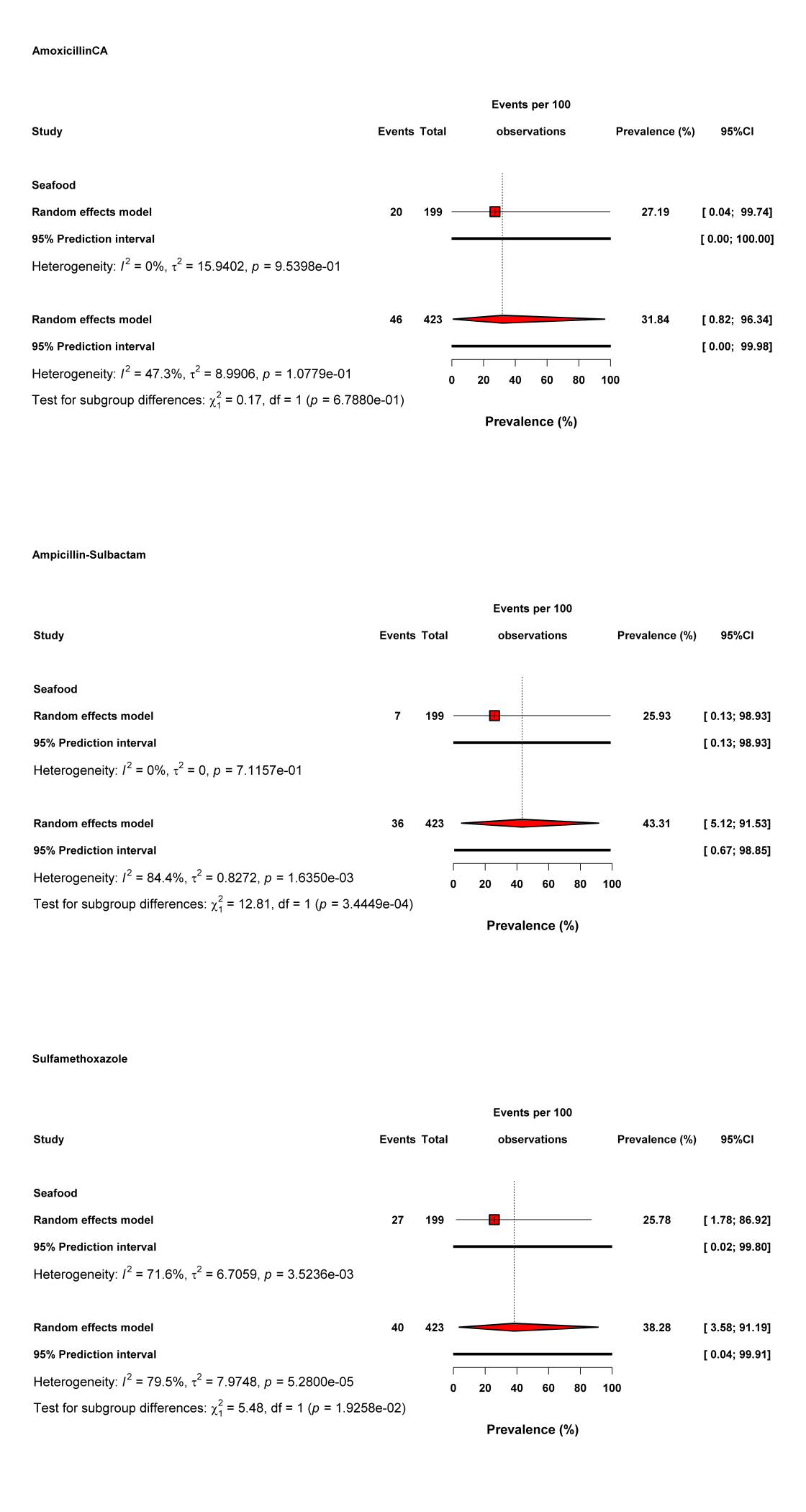

Supplement: Supplementary file 1 [file antibiotics-14-01075-s001.zip › FigureS7.tiff]

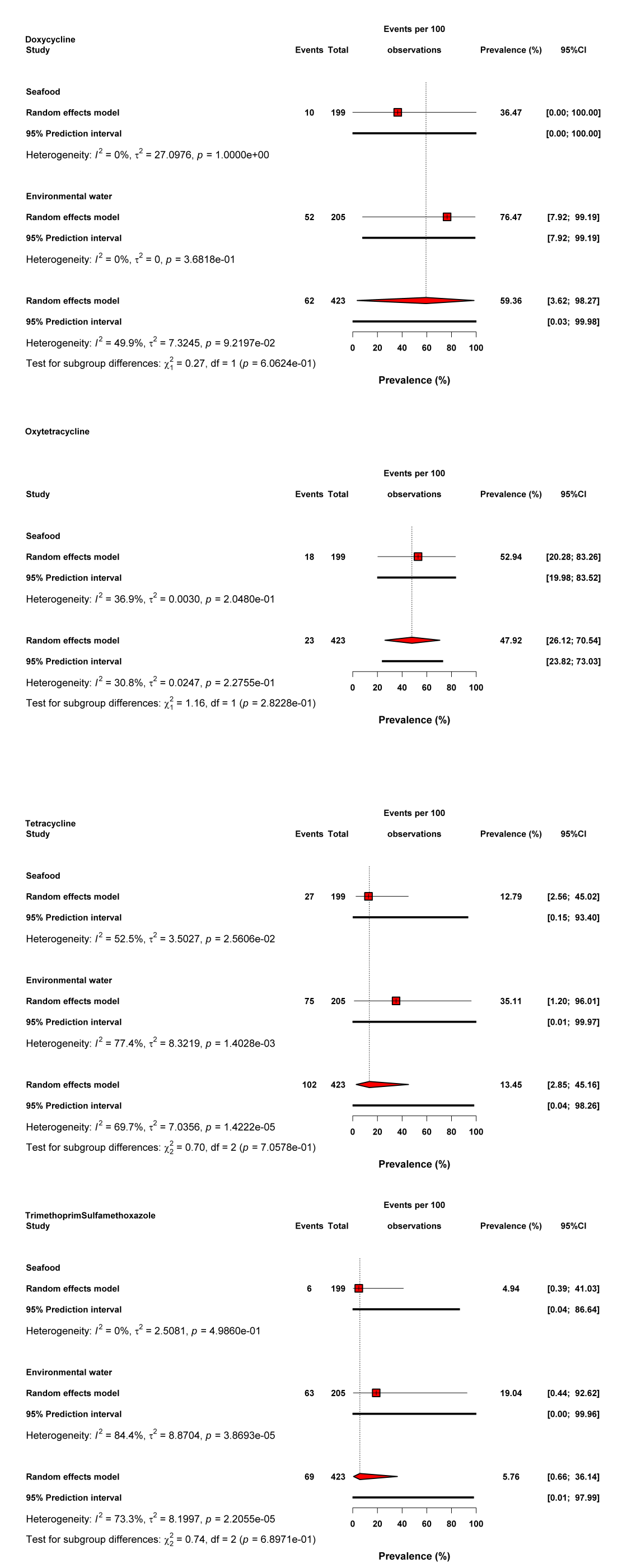

Supplement: Supplementary file 1 [file antibiotics-14-01075-s001.zip › FigureS8_Tetracyclines_sxt_4.tiff]
